# Linkage of Routinely-Collected Maternity Data with a Cerebral Palsy Register: Matching Success and Temporal Assessment of Data Quality and Maternal Characteristics.

**DOI:** 10.23889/ijpds.v7i3.1913

**Published:** 2022-08-25

**Authors:** Lisa Kent, Claire Kerr, Karen McConnell, Oliver Perra, Kelly-Ann Eastwood

**Affiliations:** 1 Centre for Public Health, Queen’s University Belfast; 2 Northern Ireland Cerebral Palsy Register, School of Nursing and Midwifery, Queen’s University Belfast; 3 School of Health Sciences, Ulster University; 4 Queen’s University Belfast; 5 Fetal Medicine, University Hospitals Bristol & Weston NHS Foundation Trust

## Objectives

This study aimed to 1) establish the success rate of matching offspring in the Northern Ireland Cerebral Palsy Register (NICPR) to mother-infant pairs within the Northern Ireland Maternity System (NIMATS); and explore changes in 2) quality of maternal data (NIMATS) and 3) maternal characteristics over the study period.

## Approach

Data for individuals with cerebral palsy (CP) born between 1990 (commencement of NIMATS) and 2012 (latest fully validated birth year in NICPR) were transferred to the Honest Broker Service, who performed data linkage. Offspring were primarily matched between NICPR and NIMATS using the unique Health and Care Number (HCN). Where this was unavailable in NIMATS, matching from offspring to mothers/pregnancies was performed using surname, gender, date of birth +/- postcode. Changes in completeness and quality of data and maternal characteristics were explored per year and visualised using histograms. All pre-processing and analyses were performed using R.

## Results

From 1263 included CP cases, 377(30%) were matched using HCN. A further 121(9%) were matched by secondary strategies. Substantial improvements in completeness of maternal data were observed over time (body mass index (46.2% to 97.7%); BP (44.8% to 95.3%); age 100% complete throughout). An apparent tendency to round systolic and diastolic blood pressure to the nearest ten was observed. This tendency decreased over time which potentially led to an overestimation of hypertension detected in earlier years. Characteristics of mothers also changed over the study duration, seen in increasing proportions with BMI≥30 kg/m2 (9.97% to 18.9%). Age of mothers also increased with decreasing proportions aged<25y (33.7% to 19.6%) and increasing proportions aged≥35y (9.7% to 20.4%). No change over time was observed in maternal blood pressure variables.

## Conclusion

Matching success was affected by incomplete NIMATS coverage in earlier years and lag time in CP diagnosis being confirmed by NICPR (typically beyond the age of 4 years). Over time, completeness and quality of maternal variables in NIMATS has improved meaning future studies using these linkages will be more reliable.

